# Foreign

**Published:** 1840-08-15

**Authors:** 


### FOREIGN.

### Russian Practice.

In a late number of the *Zeitschrift für die gesammte Medicin,* we find an abstract of a Russian Medical journal for the year 1838. The German translator has very properly omitted all that Dr. Grum, the Russian editor, had translated from foreign journals, and has also abridged the original matter. The following is a part of what he has retained.

*Cure of old ulcers.*—Dr. Schreiber, chief physician to the Brestlitoffski military hospital, gives a number of cases where old ulcers were rapidly healed by the application of fresh soft cheese; the slices being renewed every two hours. This application diminishes the pain and fœtor at the same time, and has succeeded in cases where the ordinary pharmaceutical remedies had been used in vain.

*On the external use of living ants, (formica rufa), by Dr. Schreiber.*

The summer division of the author's hospital lies in a wood, where there are so many ant-hills, that the thought struck him of drawing some advantage from them for his patients. As ant-baths and tincture of ants were of no great use, he tried the living insects in paralysis, hemiplegia, paresis, and inveterate arthritis. The ants are to be taken directly from their hill, and put in a bag; and this bag is to be tied over the limb in such a manner that the ants cannot escape [but obtain access to the skin.] Some time after their application to the paralyzed limb, the patient begins to feel the running and biting of the ants, by which they gradually excite a kind of electrical twitches, and a feeling of warmth, which gradually extends over the whole body. Moreover, by their ethereal principle, they cause as violent a perspiration over the whole body, as if the patient were in a vapour bath. The paralyzed part must be kept in the bag with the ants for two or three days; the patient is then to rest for a day, after which the ants are to be applied again; and this is to repeated till the object is attained. In 1835, Dr. Schreiber obtained a favourable result in seven cases of paralysis; in 1836, in four; and in 1837, in three; by which he was encouraged to use the same remedy in chronic rheumatism and gout. It is unnecessary to remark that this remedy alone cannot be of much service, if the case is complicated with syphilis, scurvy, or scrofula; but the military hospitals have plenty of uncomplicated cases, and in three years forty-six patients under this head were cured.

Dr. Schreiber now began to use the remedy in dropsy proceeding from inactivity of the skin. In anasarca it was found sufficient to tie up the lower extremities in bags with living ants, and thus obtain profuse sweating and a cure. This method of treatment, supported by gentle purgatives and sudorifics, succeeded in twenty-one cases. (The German translator here remarks that he cannot call ten grains of jalap with the same quantity of calomel, a "gentle purgative.")

In a case resembling elephantiasis, where the leg was cold, hard, and swollen, and a variety of treatment had been employed in vain, the disease was entirely removed within twenty days, under the use of living ants, and the patient was dismissed cured. The author mentions incidentally, that, in Little Russia, they employ a home-made spirit of ants, called *muraschkowka*, to prepare a punch which is used in many varieties of cold, with very great advantage.

*Russian Baths, as remedies against the Bite of Mad Beasts.*

As soon as the patient is admitted into the hospital, Dr. Miroff directs him to be placed in a Russian bath, and exposed for an hour, with the wound uncovered, to a temperature of 50°*. As soon as he is in the bath he drinks a pint and a half of a decoction, made with equal parts of sarsaparilla and guaiacum wood. While in the bath the wound is rubbed with mercurial ointment, and is afterwards kept open for two months by irritating ointments. The baths are at first repeated every other day, during the second and third week every three days, and afterwards twice a week, until the expiration of two months from the beginning of the treatment. Light diet is the only additional point to be observed during its continuance. The patient remains under superintendence during the third month. The author gives five histories of cases occurring at different times, and all terminating favourably.

*Allium ursinum in Scurvy.*

The efficacy of this remedy is known throughout all Grusia; but as it is to be obtained in a few places only, and soon spoils, and, when fresh, suffers much from carriage, it has been tried to preserve it acidulously,† and then eat it with vinegar. But the remedy, when preserved in this manner, not only obtained a peculiar sharpness and unpleasantness, but also lost its specific effect. Dr. Miroff, therefore, attempted to dry it carefully, which succeeded beyond his expectations. The remedy not only loses none of its efficacy, but is diminished nine-tenths in weight, making it more convenient for carriage. He gave the dried plant in scurvy with advantage, mingled with the patient's food.

* If the centigrade thermometer is meant, this will be equivalent to 122 deg. of Fahrenheit; if Reaumur's, it will be equal to 144½ deg. of Fah. It is hardly necessary to add, that the vapour-bath is intended.—Translator's Note.

† In the original, *es zu sauern*, to sour it; that is, probably, to preserve it after the manner of sourcrout.—Translator.

*Delirium Tremens from Debauchery.*

Dr. Bileff, after narrating the history of the case at great length, gives the effective prescription, which was as follows:—

℞. Morph. Acet. gr. j.; Mosch. Opt gr. iij.; Cal. gr. xij.; Sacch. ʒj. M. disp. tal. dos. No. vj.

The patient took one of these powders every two hours, and had five in all.

The German translator complains of this formula as too complicated, and thinks that if the morphia had been given alone, it might have done its duty earlier than in combination with twelve grains of calomel in each dose. So large a quantity of calomel every two hours seems the more dangerous, he adds, as the patient had already sunk very low through continual drunkenness, syphilis, and the frequent use of mercury, and, therefore, had not much more strength to lose.

*Practical Remarks, by Dr. Krassnoglädoff, of the village of Suchadi, in Mingrelia.*

1. He directs our attention to the affections of the spinal marrow in intermittent fever, which so commonly occur in Mingrelia. The complaints of the patient of pain in the back during the fit are at first commonly disregarded; but when they occur, bark does not cure the case, and then the practitioner begins to pay greater regard to this symptom in the following paroxysms. Moreover, when the patient, after the paroxysm, complains of a severe sensation of being bruised, and paralysis of the upper or lower extremities, the corresponding part of the spinal marrow is affected, a fact which is clearly ascertained on examination. Blisters on the part soon relieve the patient, and eight grains of quinine will now cure an intermittent, where a drachm between two paroxysms had previously been of no avail.

2. As the itch insect, and its propagation under the skin, have been demonstrated, Dr. Krassnoglädoff proposes the question, whether these animalculæ are not able to enter the body, live in the alimentary canal, and multiply there, as well as under the skin? This question is prompted by the following case: "In August, 1837, a peasant was brought to me from a village in Mingrelia. He had had the itch for several years, which is extremely common among his country people, until it covered the whole surface of his body. It is unnecessary to mention, that the head, palms of the hands, and soles of the feet, were free from pustules; but, with these exceptions, no part of the body had remained unscathed, only that there were vacant spaces between the clusters on the chest and abdomen. The patient could not do his work, but went to bed at noon, in order to mitigate the itching by scratching. But it was not the external eruption that annoyed him so much. In the thorax, along the course of the œsophagus, and in the direction of the stomach, he had an unceasing and an unbearable itching, which gave him the most uneasiness. I employed the English method of treating itch, and prescribed flowers of sulphur internally, adding a drop of oil of lavender to each dose. The patient also suffered much from constipation, so that frequent purgatives were necessary. The internal itching decreased with the decline of the eruption, and on the sixteenth day the patient was free from both. The internal eruption, therefore, had a close connection with the itch, and we may assume that this itching was caused by scabies, as it was removed simply by the use of a specific against that disease."

*Hydrophobia.*—At the meeting of the Society of Russian Physicians at Petersburgh, on the 20th Dec., 1837, Dr. Brikoff stated that, in the government of Koursk, the *Thalictrum flavum* (Spanish meadow-rue) is used with advantage against hydrophobia. An infusion is made with two ounces of the herb, and sufficient water to produce sixteen ounces after straining, and this quantity is taken in twenty-four hours. Dr. Grum remarks upon this, that the plant was long ago described, and that Professor Smeloffski was sent by the medical board into that country, in order to collect what was known about it on the spot.—*Lon. Med. Gaz.*

---

*Experiments on the Mechanism of Inflammation.* By Dr. Robert Latour.—M. Latour details a number of experiments which seem to demonstrate that cold-blooded animals are not susceptible of the inflammatory process; and that, consequently, the experiments of Wilson Phillip, Thompson, Hastings, Kaltenbrunner, Gendrin, and others, made on these animals, with the view of demonstrating the nature of the inflammatory process, go for nothing. M. Latour has in vain endeavoured to excite inflammatory action in carps and frogs, by applying various irritant substances to the skin, to the abdominal cavity, and to the other tissues of the body; and eighteen experiments are given in detail, in proof of his statement. The following is a short abstract of the results:—

Deep incisions were made through the skin and muscular fibre of carps and frogs, and the wounds afterwards stimulated by the application of various acrid agents; as ammonia, concentrated acids, &c., which, though they caused great pain, were not followed by any reaction, either local or general. A peg of wood remained three days in the muscular fibres of a carp; pins were left several days transfixing the limbs of frogs; a piece of wood was left for thirty-six hours in contact with the mesentery of a frog; a piece of a potato was for a much longer time within the abdominal cavity of another; and a seton was kept in the thigh of the same animal for more than a month; the skin of a carp was cauterized with sulphuric acid; a red hot iron was applied to the thigh of a frog;—and yet in not one of these numerous and oft-repeated experiments, could the least appearance of inflammatory action be traced in the irritated wound; there was neither redness, swelling, sanguineous injection, nor suppuration. Nay, more, in those experiments where the muscular fibre of the animal was exposed, the redness which came on immediately after the incision was made, disappeared, and the wound filled with a glutinous matter, which served as the basis of the cicatrix. These animals were not, however, insensible to pain, as they showed by their writhings how much they suffered from the operations.

These results being in direct opposition to those arrived at by other experimentalists, M. Latour endeavoured to find out the cause of the difference. As it was chiefly on the frog that former physiologists had experimented, and as it was from the phenomena observed in it that they announced they had developed inflammatory action, he repeated their experiments, and succeeded in producing a sanguineous injection, very visible to the naked eye, on the upper and inner surface of the thigh of a frog, by pinching it with a pair of forceps previously dipped in ammonia. He remarked, however, that these parts became covered with a tolerably abundant viscous sanguinolent secretion, and that the animal was seized with convulsions, and expired. M. Latour then discovered that the blood had been coagulated by the action of the ammonia, after having parted with its fluid parts, in precisely the same manner as happens to the blood of the frog when acted on by ammonia out of the body; as soon as the two liquids come in contact they lose their fluidity, and become a homogeneous blackish mass, of a plastic nature, and not easily broken up.

M. Latour inferred from this, that the redness caused by the ammonia was not of an inflammatory nature, but that it was the effect of the decomposition of the blood, which, being deprived of its fluid portions by its affinity for the alkali, was consequently stopped in its circulation by its coagulating within the vessels.

When a frog was plunged in salt water the skin was reddened, and the animal killed. In this case the blood was also decomposed, became more fluid, and the colouring matter was precipitated; and it is to this circumstance that M. Latour attributes the increased redness of the skin. The same peculiar colour was produced by mixing the blood of the frog with a solution of sea-salt.

Similar results followed the application of a red hot iron to the webbed foot of the frog, when placed in the focus of a microscope. As the red hot iron was brought nearer and nearer to the web, the blood became coagulated first in the smaller vessels, and afterwards in those of a greater volume.

These facts throw doubts on all the conclusions which have been drawn from the observations of experimentalists, on the excited vascular actions of the cold-blooded animals. M. Latour is inclined to the belief, that inflammatory action can only exist in those classes of animals where the temperature of the body is constantly in an exalted condition; in fact, in the warm-blooded animals alone. Many of his opinions are too fanciful, and rest on too insufficient data to be further noticed.—*Edinburgh Med. and Surg. Journ.*, *from Revue Medicale*, Jan. 1840.

*Lactate of Iron in Chlorosis.*—The pastilles of the lactate of iron have been of late strongly recommended in the treatment of chlorosis. MM. Bouillaud and Fouquier have made numerous trials of it in this disease, and it appears to have surpassed their most sanguine expectations. They describe it as causing increase of the appetite the first days of its employment, soon followed by cessation of the symptoms of chlorosis; and a cure has generally been effected in from fifteen to twenty days.—*Ibid.*, *from Archives Generales de Medecine*, March, 1840.

*New Facts with regard to Strangulated Hernia.* By M. Laugier.—Every one knows that in strangulated hernia, the upper extremity of the intestine, or that part which is comprised between the stomach and the seat of strangulation, becomes distended by fluid, gaseous, or fecal matters, whilst the lower portion remains flaccid, or, at least, the matters found in it do not distend it more than usual.

No attempt has yet been made to draw practical inferences from this fact, so as to use it as a means of distinguishing intestinal from omental hernia; or which portion of the intestinal tube, whether the large or small intestine, was contained in the hernial sac. This has, however, been done by M. Laugier, who considers it both as a simple and sure means of diagnosis, besides possessing the additional advantage of indicating the relative urgency of the case for operation. The results of his observation are embodied in the following conclusions.

1. The extent, the form, and seat of the meteorism of the belly, during strangulated hernia, vary according as the hernia consists of omentum or intestine, and according as the upper extremity consists of the small or the large intestine. Its extent varies also according as the portion of small intestine included in the hernia is more or less distant from the stomach.

2. In omental hernia, before the developement of peritonitis, the belly is soft, and flat, even in the neighborhood of the hernia. There is no meteorismus, (windy swelling.)

3. Meteorismus appears very rapidly when the hernia consists of intestine.

4. When the hernial sac contains the large intestine, the sigmoid flexure of the colon, for example, the upper extremity consisting of nearly the whole of the intestinal tube, the meteorismus is general, and gives to the abdomen an almost cylindrical form.

5. If the small intestine is alone in the hernial sac, or along with a portion of omentum, the flakes and the epigastric region are soft and depressed; the swelling of the abdomen occupies the hypogastric and umbilical regions. The swelling is spherical, or nearly so, resembling in this respect some encysted tumours, or the uterus at the sixth or seventh month of pregnancy, from which, however, it differs in many essential characters.

6. When the strangulated part of the intestine is near the stomach, the swelling of the abdomen is trivial in proportion to the duration of the strangulation; for in that case the upper extremity of the tube is only of small extent.

7. From these facts may be deduced the unexpected and important conclusion, that in crural hernia, and in some inguinal hernias, where gangrene is most to be feared, all haste should be used either to reduce it by the taxis or by operation; for if by the occurrence of gangrene of the intestine an artificial anus should be formed, its proximity to the stomach renders it more dangerous for the patient. A swelling of small extent, limited to the neighbourhood of the hernia generally, inspires a security which may lead to fatal results; whilst it is the general meteorismus, or one, at least, which is very extensive, that alone permits of delay, since it indicates the incarceration of a portion of intestine which is far from the stomach.

8. It is only before the accession of general peritonitis that the characters of the windy swelling, above indicated, can be relied on; they are most marked at that stage.

M. Laugier adds, that these remarks have been all verified by careful clinical observation, and by the result of many operations for hernia.—*Ibid.*, *from Comptes Rendus*, 2d March, 1840.

*New Method of treating Retroversion of the Uterus.* By Charles Halpin, Esqr.—Mr. Halpin was called to attend a case of retroversion of the uterus, in a woman of about 23 years of age, and between the fourth and fifth month of her pregnancy. The abdomen was greatly distended from an accumulation of urine in the bladder, and for the last two days before being seen she had not passed more than two ounces of urine. On examining by the vagina, the uterus was discovered to be completely retroverted, the fundus lying low in the pelvis, within an inch of the external parts. After several vain attempts to introduce a catheter, Mr. Halpin at length succeeded, and drew off, by means of a flexible male catheter, several pounds of urine. The urine was drawn off in this manner for several days, and various attempts were made to restore the uterus to its normal position, but in vain; and as the symptoms were becoming every day more aggravated, it was quite apparent that some plan, besides those usually recommended in retroverted uterus, must be had recourse to in order to give relief.

In attempting to replace the uterus, Mr. Halpin found that he could always move it to a certain extent, but that after it reached this, it became fixed, and he could feel the parietes of the uterus yielding under the point of contact of the fingers. He therefore inferred that the only chance of rescuing the patient from her perilous state, would be in the use of some instrument which could be brought to bear equally on all parts of the tumour, and with which sufficient power could be applied to raise it fairly above the promontory of the sacrum.

It occurred to him, that with the assistance of a bladder he would be able to inflate the pelvis, and thus raise its contents into the abdomen; and he immediately acted on this suggestion. He attached a small recent bladder to the tube of a stomach-pump, with an air-tight piston, and having immersed it for a few moments in warm water to bring it to the temperature of the patient's body, he introduced it empty into the vagina, between the fundus of the uterus and the rectum. It was retained in this position, by the hand being pressed firmly over the orifice of the vagina, and cautiously inflated. After a time, the patient complained of a sensation of tension or bursting, but not of pain. He then ceased throwing more air into the bladder for about five minutes, allowing what was in already to keep up a steady, well-directed pressure on the tumuur. At the expiration of the five minutes, more air was thrown into the bladder, when the patient exclaimed that he was forcing something up to her stomach. The bladder was retained for some little time, and then a small portion of air was allowed to escape from it, so as to permit of the introduction of a finger, when he had the satisfaction to find that the tumour was no longer to be felt in the pelvis, and that the *os uteri* lay within reach of the finger, and pointed downwards and backwards. Upon this the apparatus was withdrawn.

The simplicity and safety of this apparatus recommend it strongly to the obstetrical practitioner, and it assuredly appears better fitted for accomplishing the end in view, viz. the restoration of the uterus to its natural position, than any of the other painful methods which have been proposed. Mr. Halpin suggests, that if the introduction of air should not be found to give a sufficient dilating force, water could be easily employed, which, at the same time that it puts us in possession of an irresistible power, is so completely under control that no bad consequences can result.

Mr. Halpin recommends the same instrument to be used as a pessary for the retaining of the uterus in its position after its replacement. Being filled with air it will occasion less annoyance to the urinary passages, and cannot, from its nature, obstruct the free passage of the urine or fæces.—*Ibid.*, *from Dublin Journal of Medical Science*, March, 1840.

---

*Puncture of the Bladder a safe operation.*—M. Levrat of Lyons was lately called to a child labouring under retention of urine, caused by a urinary calculus lodging in the urethral canal. To relieve the present urgent symptoms, he punctured the bladder, and afterwards broke down the calculus in the urethra, and extracted it; the child rapidly recovered. M. Levrat has frequently punctured the bladder, and in every case the operation was followed by the happiest results.

M. Gerder regards puncture of the bladder as a very safe operation. He has performed the operation in various cases and circumstances, and always successfully. One of his most interesting cases was one where the perineum was much bruised in consequence of a fall on that part: retention of urine was the consequence; puncture of the bladder was performed, and the patient rapidly recovered.—*Ib.*, *from Seances de l'Academie Royale de Medecine*, 14th January, 1840.

---

*Case of Poisoning by Colchicum Seeds.* By Dr. Neubrandt.—A man, 52 years of age, took by mistake, on the evening of the 18th of February, a decoction of the seeds of colchicum, made with a table spoonful of the seeds to a pound and a half of water. During the night he vomited and passed more than fifteen stools. He was seen by Dr. Neubrandt next morning, but was not then complaining much. The vomiting and purging were less frequent, and though he felt weak, he had no pain, and could sit up. The abdomen, though not distended, contracted spasmodically when touched; the pulse was small and rather quick; the stools had a very fetid odour, and contained a quantity of whitish looking membranes. He was first ordered tepid water and melted butter, but this excited the vomiting and purging of new. Strong infusion of coffee, and also infusion of marsh mallows with lemon juice was next prescribed.

Next morning, the 20th, he presented the following symptoms: his face was pale, the eyes sunk in the orbits, and the pupils very much dilated, the respiration was hurried, and he groaned much. The tongue was covered with whitish fur, and was with great difficulty protruded. The region of the stomach was painful. His breath, face, and extremities, were cold; the pulse very quick, and scarcely to be felt. The purging was more constant, and the stools contained matters of a clear blue colour, in considerable quantity. Although he gave distinct answers to questions which were put to him, his intellectual faculties seemed to be confused. He died at ten o'clock. The only symptoms of any portion of the poison having been absorbed, were the continuance of the vomiting and purging.

The body was examined twenty-three hours after death. The pupils of the eyes were very much dilated, the eyes were much sunk, and the mouth spasmodically closed. There was remarkable rigidity of all the members and muscles. The abdomen, scarcely more swollen than during life, was of extraordinary rigidity, and was covered with peculiar looking spots, which were especially numerous in the region of the stomach, and on the sides of the abdomen; these spots were of a violet and greenish blue colour, presented the appearance of rays, and were not circumscribed. The muscles were of a deep blue colour, as if they had been dried in the air. The trachea was inflamed at its bifurcation. The lungs were collapsed, pale, and soft to the touch, but not diseased. The heart was turgid with coagulated blood, and covered with spots of a black, violet, and brown colour. The œsophagus was of a natural appearance till towards the cardia. The stomach was of a light violet colour, its cardiac orifice being of a deep violet. The veins of both stomach and intestines were much distended by very dark blood. The liver had a violet hue over its concave surface; and the gall-bladder was much distended with green bile. A few red and brown mottlings were observed on the surface of the intestines, but otherwise they presented nothing unnatural. The other organs of the body appeared to be healthy.—*Ibid.*, *from Mediciniscbes Correspondenz-blatt*, 1840.

---

*On the Plague of Egypt.* By M. Clot-bey.*

When I left France for Egypt, in the year 1825, my ideas on the nature of the plague were entirely derived from writers on that disease. Like them, I, also, was a contagionist; and my opinion was still further confirmed by a perusal of the works of the medical officers attached to the expedition into Egypt. I even went so far as to provide myself with a long stethoscope, for the purpose of examining the pulse of persons labouring under the plague.

On the day after my arrival, (Feb. 11, 1825) a ship captain requested me to visit a patient whom he supposed to be attacked by some malignant fever. I found the man in a state resembling that produced by a drunken debauch; his countenance was sunken, the eyes injected, and the gait unsteady. He complained of pain in the shoulder, where there was an enormous slough, and the axillary glands were enlarged. The analogy between these symptoms and those of the plague, struck me; but the vessel came from Cyprus, where no case of plague existed at the period of its departure. The French consul-general laughed at my suspicion; but on the patient being examined by some Egyptian physicians, it was discovered that he really laboured under the plague.

This case made a deep impression on my mind. Myself, the captain, and several other persons, had touched the sick man, and afterwards held free communication with the people on shore; yet, although the patient died of the disease, not a single individual caught it from him. I soon took my departure for Cairo, and there observed two cases of plague during the year 1825. I instituted the most careful inquiries, but could not discover any thing calculated to confirm my preconceived ideas respecting the contagious nature of the plague. In the year 1832, I returned to France. Dupuytren required of me some information, and requested to know my opinion on the contagious or non-contagious nature of the disease. I was unable to give a satisfactory answer, because the facts which I had observed were too few in number to justify my drawing any decided conclusion from them. These few remarks show that my mind was completely free from prejudice at the period when the dreadful epidemic of 1835 set in. As chief of the medical staff, it became my duty to advise the government, and ordain all the necessary measures during the prevalence of the epidemic, which lasted five months. The result of the experience then obtained I shall now communicate, and in particular direct your attention to some points connected with the origin, etiology, treatment, and contagious or non-contagious nature of the plague.

*Origin of the Plague.*—The majority of writers assert, that the plague has existed in Egypt from time immemorial; but recently, a few medical men have put forth a contrary opinion. A commission of French physicians was sent into Egypt to decide the question, and came to the conclusion that the plague was a disease of recent origin. Yet this opinion admits of reasonable doubt. We may even hesitate to admit that any epidemic disease is of recent origin. The cholera has always existed in India; and measles, small-pox, scarlatina, &c., have certainly prevailed from time immemorial. But what reason have we for supposing the developement of a new epidemic? Has the climate changed? Has the nature of the soil undergone any change sufficient to explain the nature of a new disease? The advocates of contagion are accustomed to bring forward the fact of embalming as a proof that the Egyptians were, in former times, aware of the possibility of the plague being communicated by exhalations from the body. But were this the case, why did they neglect to embalm their slave population? All the circumstances which attended embalming prove that it was a religious ceremony, and not a measure connected with public health. Hence, I conclude

---

* The substance of a lecture delivered by M. Clot-Bey, to a numerous assemblage of medical men, on the 26th of April last, in the lecture room of La Pitié.

that plague has existed in Egypt from time immemorial.

*Etiology of the Plague.*—The immediate cause of plague is as little known to us, as is that of cholera, yellow fever, scarlatina, or the influenza, &c. We merely perceive some connection between its spread and the medical constitution of certain periods of time. Writers, however, persist in attributing the disease to particular causes. Thus, some will attribute it to the overflow of the Nile; but they are evidently ignorant of the nature of this overflow. The residue which is left by the Nile is nothing but pure earthy soil, without any mixture of vegetable or animal matter; the layer deposited is not much thicker than a sheet of paper, and it is even doubtful whether the irrigation be favourable to vegetation, so small is the quantity of alluvial matter deposited by the waters. Other writers speak of evaporation or exhalations from the Nile, which brings with it decayed vegetable matter from the marshes traversed during its course. Were this the case, plague should be most prevalent in Upper Egypt, where such marshes exist, and in Nubia; but it is almost unknown in those countries. The truth is, that very false ideas prevail concerning the overflow of the Nile. The inundation of Egypt is completely artificial, and takes place only when it is thought advisable to effect it by opening the dykes. Upper Egypt is first irrigated; then Nubia; then Lower Egypt. Any extensive inundation is the result of accident; but even then it has not been observed that plague prevails more extensively, or with greater intensity, than at other times. Various other causes have been assigned, such as poverty, filthiness, of the inhabitants, &c. To these a simple answer suffices: they exist in much greater force in Upper Egypt, where, as I have remarked, plague is unknown. Hence, I conclude that local causes have no influence in the production of plague.

*Symptoms.*—The want of time does not permit me to enter upon this point. You will find the symptoms of plague perfectly described in books.

*Treatment.*—The remark which every scientific physician has applied to cholera, is equally applicable to plague. The whole range of the materia medica, every known therapeutic agent, has been tried over and over again, without success. When any morbific agent attacks the system with such violence, as, within the space of three days, to quadruple the size of the spleen, to render the mesenteric glands as large as oranges, while at the same time it reduces them to a mere jelly, to force the blood from its vessels, &c., what hope can we place in medicine? How difficult must it be to arrest such fearful destruction?

*Morbid Anatomy of the Plague.*—On this I should wish to dwell at some length, but time does not permit me to do so. I shall merely observe, that a few bodies were examined by the French commission; but the abdominal cavity alone was examined, and that in a superficial manner: hence the morbid anatomy of plague was imperfectly understood until the last few years. It would detain me too long to place before you a faithful detail of the pathological appearances; I shall, therefore, defer the consideration of this point to some future opportunity, and pass to an examination

*Of the Contagious or Non-contagious Nature of the Plague.*

This, perhaps, is the most interesting point in the history of the plague: it was never examined by the ancients. The moderns admit two forms of contagion: one by miasma, the other by inoculation. Let us see whether the plague belongs to either of these two forms. We cannot establish the existence of a virus capable of being propagated by inoculation. Where are the external characters which constantly exist in all diseases depending on a virus? Not the malignant pustule, which exists only in one-third of the cases. Not the bubo, which is far from being a constant symptom. Finally, the matter of plague-bubo, and of the malignant pustule, does not produce plague, when introduced into the system by inoculation. Hence, we reject the idea of contagion by virus.

*Contagion by Miasma.*—This has given rise to the theory of infection. The advocates of this theory say, that the first person who was attacked by plague must necessarily have acquired the disease under the influence of general causes, as he could not have caught it from any other individual. These general causes are the decomposition of animal and vegetable matter. Let us admit this theory for an instant: the vehicle of the miasma must be the atmosphere; hence, any attempt to avoid the exciting cause, or miasma, must be vain, and the sanatory measures now adopted, useless. But we have other objections of a more serious nature. No one knows how the epidemic commences; here the contagionists are sorely embarrassed; the disease passes through its different phases and disappears. The dead bodies of its victims remain, their clothes are worn by the survivors, but no fresh case appears after the months of May and June. During the epidemic at Alexandria, from five to six hundred houses, in the quarter where plague broke out, were shut up by order of the government; the doors and windows were built up, and the keys deposited in the hands of the authorities. On the cessation of the epidemic, these houses were thrown open; yet, although the air had remained unchanged, and consequently charged with miasmatic poison, (had any such existed) the persons who occupied the houses were not attacked by plague; the furniture, clothes, &c., contained in the condemned houses, were sold by public auction, yet not a single individual amongst buyers or sellers was affected by the supposed contagion.

Here is another of a similar nature: In the hospitals the same clothing was distributed, during the epidemic, to various classes of patients; and, after its cessation, the pestiferous garments, impregnated with the matter of buboes, pustules, &c., were given to the ordinary patients, yet not one case of plague was occasioned by this practice. From the above considerations, I conclude that plague is an epidemic disease analogous to cholera, yellow fever, &c., and dependent on some cause which is different from contagion. It is unnecessary, then, to say, that I regard quarantine as altogether useless. Besides, it is a measure completely delusive, being violated in every possible kind of way. I have seen occurrences take place at Marseilles of the most extraordinary kind, when we consider that the lazaretto is directed by the most strenuous advocates of contagion. While I was detained there myself, in quarantine, I passed a carpet and a Cachmire shawl; and similar infractions are daily committed in every quarantine station throughout Europe. History, also, shows how ill-founded are the fears which prevail concerning the propagation of plague by contagion. The Crusades never occasioned any extension of the disease from east to west; and the actual deaths produced by plague were less before the institution of lazarettoes than they have been since. It is said that Europeans escape the plague, in consequence of the precautions which they take to avoid contagion. I can assure you that this is false. The most perfect seclusion is no guarantee. As a proof of this, I shall relate to you the most striking fact with which I am acquainted. On the outbreak of the epidemic, the Pacha retired to a garden, at some distance from Alexandria, situate in a most delightful and healthy locality; the garden was surrounded by a wooden palisade, and outside this was drawn a military cordon, the sentinels being placed at distances of fifty paces from each other; yet a negro servant was suddenly attacked by plague, and the disease extended to seven other persons. Four of the military were attacked at a subsequent period. —*London Lancet, from French Lancet,* April 28, 1840.

---

*New Remedy for Tetanus and other Convulsive Disorders.* By W. B. O'Shaughnessy, M. D., Calcutta.—The following particulars concerning the efficacy of a new remedy for tetanus, discovered by Dr. O'Shaughnessy, are contained in the last number of the "British and Foreign Medical Review."

The narcotic and intoxicating effects of hemp are popularly known in various parts of Asia, Africa, and America, where it is extensively employed in a multitude of affections; but in Western Europe the use of hemp is unknown either as a stimulant or as a remedy, probably because the European hemp does not contain any of the resinous matter upon which its therapeutical properties depend. In warm climates, and during certain seasons, a resinous juice exudes and concretes on the leaves, slender stems, and flowers of the hemp. This resin has a fragrant, narcotic odour; bitter, acrid taste; and, when pure, is of a blackish gray colour. It is very soluble in alcohol, or fixed oils, and is insoluble in acids.

Having determined, by experiments on carniverous animals, that the remedy might be administered with safety to the human subject, Dr. O'Shaughnessy proceeded to try its effects in several convulsive diseases. The resinous *extract* which he employed, was obtained by boiling the tops of the dried hemp plant in spirit, (sp. gr. 835,) until the resin was dissolved, and then evaporating the tincture to dryness. The tincture of hemp was prepared by dissolving three grains of this extract in one drachm of proof spirit. The doses vary according to the disease for the cure of which the remedy may be employed. In cholera, Dr. O'Shaughnessy gives ten drops of the tincture every half hour, until the vomiting and purging are allayed. In cases of tetanus, a drachm of the tincture every half hour, until the paroxysms cease, or catalepsy is induced. In hydrophobia, ten or twenty grains of the extract to be chewed by the patient, and repeated according to their effect.

The diseases for which the hemp resin has been administered by Dr. O'Shaughnessy, are rheumatism, cholera, hydrophobia, and tetanus. In the two former complaints, the trials hitherto made do not lead to any determinate conclusion. In one undoubted case of hydrophobia, the effects of the resin are thus graphically described by Dr. O'Shaughnessy:

"By his own desire, water was brought in a metallic vessel, which he grasped and brought near his lips: never can I forget the indescribable horrors of the paroxysm which ensued. It abated in about three minutes, and morbid thirst still goading the unhappy man, he besought his servant to apply a moistened cloth to his lips. Intelligent and brave, he determinately awaited the contact of the cloth, and for a few seconds, though in apalling agony, permitted some drops to trickle on his tongue; but then ensued a second struggle, which, with a due share of the callousness of my profession, I could not stand by to contemplate. Two grains of hemp resin, in a soft pillular mass, were ordered every hour; after the third dose, he stated that he felt commencing intoxication; he now chatted cheerfully on his case, and displayed great intelligence and experience in the treatment of the very disease with which he was visited. He talked calmly of drinking, but said it was in vain to try, but he could suck an orange; this was brought to him, and he succeeded in swallowing the juice without any difficulty. The hemp was continued till the sixth dose, when he fell asleep, and had some hours' rest. Early the ensuing morning, however, Mr. Siddons, my assistant, was called up to him, and found him in a state of tumultuous agony and excitement. The hemp was again repeated, and again by the third dose the cheering alleviation of the previous day was witnessed. He ate a piece of sugar-cane, and again swallowed the juice; he partook freely of some moistened rice, and permitted a purgative enema to be administered. His pulse was nearly natural, the skin natural in every respect. His countenance was happy.

"Four days thus passed away, the doses of hemp being continued. When he fell asleep, on waking the paroxysms returned, but were again almost immediately assuaged as at first. Meanwhile, purgative enemata were employed, and he partook freely of solid food, and once drank water without the least suffering. But about three P. M. of the fifth day, he sunk into a profound stupor, the breathing slightly stertorous; in this state he continued, and without further struggle, death terminated his sufferings at four A. M., on the 27th of November."

In several cases of traumatic tetanus, the power of the remedy was triumphantly exhibited. In the first case, symptoms of tetanus supervened on the employment of a moxa, for the cure of dysentery. Two days after their appearance, the case was considered hopeless, and the extract of hemp was administered, in the dose of two or three grains, every third, and then every second hour. The spasms were speedily mitigated, and ceased altogether in 11 days. The dysentery proved fatal, however, seventeen days afterwards. The other cases are thus alluded to by Dr. O'Shaughnessy:

"The *second case* was that of Chunoo Syce, in whom tetanus supervened on the 11th of December, after an injury from the kick of a horse. After an ineffectual trial of turpentine and castor oil in large doses, two-grain doses of hemp resin were given on the 26th of November. He consumed in all, 134 grains of the resin, and left the hospital cured, on the 28th of December.

"*Third Case.*—Huroo, a female, ætat. 25, admitted into the native hospital, December 16th, had tetanus for three previous days, the sequel of a cut on the left elbow, received a fortnight before. Symptoms violent on admission. Turpentine and castor oil given repeatedly without effect; on the 16th and 17th, three grains of hemp resin were given at bed-time. On the morning of the 18th she was found in a state of complete catalepsy, and remained so until evening, when she became sensible, and a tetanic paroxysm recurred. Hemp resumed, and continued in two-grain doses every fourth hour. From this time till the third hour, tetanic symptoms returned. She subsequently took a grain twice daily till the 8th February, when she left the hospital apparently quite well.

"Mr. O'Brien has since used the hemp resin in five cases, of which four were admitted in a perfectly hopeless state. He employed the remedy in *ten-grain doses*, dissolved in spirit. The effect he describes as almost immediate relaxation of the muscles and interruption of the convulsive tendency. Of Mr. O'Brien's seven cases, four have recovered.

"In the Police Hospital of Calcutta, the late Dr. Bain has used the remedy in three cases of traumatic tetanus; of these, one has died and two recovered.

"A very remarkable case has recently occurred in the practice of my cousin, Mr. Richard O'Shaughnessy. The patient was a Jew, æt. 30, attacked with tetanus during the progress of a sloughing sore of the scrotum, the sequel of a neglected hydrocele. Three-grain doses were used every second hour, with the effect of inducing intoxication and suspending the symptoms. The patient has recovered perfectly, and now enjoys excellent health."

"The preceding facts," says Dr. O'Shaughnessy, "seem unequivocally to show, that when given boldly and in large doses, the resin of hemp is capable of arresting effectually the progress of this formidable disease, and, in a large proportion of cases, of effecting a perfect cure." We trust that some of our hospital physicians will, without delay, procure the remedy which Dr. O'Shaughnessy has thus favourably introduced, and determine how far it may sustain its reputation as a "powerful anti-convulsive," in this country.—*B. and F. Review.*

---

*Emphysema of the Submucous Tissue of the Stomach.*—Dr. Hutton laid before the Pathological Society a stomach, in the submucous tissue of which air had been secreted in large quantity, elevating the mucous membrane, and forming a congeries of transparent vesicles, situated towards the greater extremities of the stomach; the lining membrane of the organ was slightly inflamed, but nowhere softened; the œsophagus superficially ulcerated, and the duodenum preternaturally vascular; the liver was hypertrophied and the kidnies in a state of congestion. The cavities of the heart were empty, and the lining membranes of the great arterial trunks were tinged of a deep scarlet colour; the left cavity of the pleura and the lungs contained dark coloured fluid blood; the brain presented nothing abnormal, with the exception of slight effusion into the ventricles. The specimen was taken from the body of a man, æt. 36, who was admitted into the Richmond Hospital, labouring under the usual symptoms of delirium tremens, but not to an alarming amount. On the evening, however, of the day after his admission, he was attacked with violent delirium, followed by rigors, cold perspirations, and convulsions, and died at twelve o'clock at night. Dr. Hutton alluded to the cases of pneumatosis of the intestinal mucous membrane, recorded by various authors, and concluded by drawing the attention of the Society to the connection that appeared to exist in such cases between the fluid state of the blood and the occurrence of emphysema in various tissues of the body.—*Dublin Journal of Medical Science.*

---

*Rupture of the Right Ventrical of the Heart.*—Mr. Smith exhibited the heart of a man, who for two years previous to his death had suffered from repeated attacks of rheumatism. He never complained of any affection of the heart until the night of the 11th November, when he was suddenly seized with symptoms of collapse, and anxiety about the præcordia; his pulse fell to forty in the minute, his extremities became cold, his countenance pale, and his whole body was bedewed with cold perspiration. He remained in this state for eighteen or nineteen hours, and died upon the 12th.—Upon examination after death, the pericardium was found distended with blood, and a small lacerated opening was seen in the apex of the right ventricle, near the septum: the parietes of the ventricle became gradually thinner towards the seat of rupture; in other respects the heart was healthy. From a table drawn up by Dr. Townsend, it appeared that of twenty-five cases of rupture of the heart, in only three was the right ventricle the seat of the rupture; and of nineteen cases collected by Bayle, but three occupied the right ventricle. Mr. Smith remarked that the case he brought forward he considered interesting from its rarity, but it derived an additional interest from the circumstance that the symptoms would lead to the supposition that the rupture had taken place eighteen hours before death: life being prolonged under such circumstances could only, he conceived, be explained upon the supposition, that a coagulum had blocked up the opening in the right ventricle: he supposed that death at length took place when the coagulum was expelled, and concluded by alluding to the cases recorded by Cruveilhier and others, where a firm fibrinous concretion plugged up the fissure.—*Ibid.*

---

*Abortion of five Fœtuses in the third month.*—Dr. E. Kennedy presented a specimen taken from a female who had borne five children at once, and had aborted in the third month of pregnancy. The specimen consisted of three distinct ova, with their appendages. Two of these were double, and contained twins, the third was single; each of the double ova were enveloped in a common membrane, and had a common placenta; the single ovum had its own placenta and membranes. Some persons were disposed to question the occurrence of these multiparian births, but there had been two or three well-authenticated instances of five children at a birth. One of these occurred lately at Naples, the other about twelve years ago in North America. It was a curious fact, that in the matter of multiparian births, Ireland preponderated. The proportion of twin cases in this country is one in sixty; in London it is one in ninety-one; in France, one in a hundred and forty; in America (where there is a large proportion of Irish settlers,) it is one in seventy-five. It was also a curious fact, that the female who gave birth to the five children at once, in America, was an Irish emigrant. The female from whom the specimen was taken aborted with bearing-down pains about the third month of her pregnancy, most probably from over distension of the uterus. She had laboured under constant nausea, a symptom which appears to be one of the most ordinary signs of multiparian pregnancy.—(Museum Lying-in Hospital.)—*Dublin Journal of Medical Science.*

---

*Interesting Case of Œsophageal Arrest of Dedevelopment.* By Thomas Warner.—On the 21st of February, J. D. was delivered of a male child, well formed externally, though small in size: the cries were as loud as is usual in new-born infants; and the first deviation from a healthy condition reported by the nurse, was a constant rejection of the food. Injections were used, and the intestines were evacuated, but still nothing appeared to pass into the stomach, although the child seemed eager for food, and applied itself to the nipple.

On watching more attentively the process of deglutition, it was evident that the attempt was immediately followed by a regurgitation of the food with saliva through the nostrils; thus clearly indicating some mechanical obstruction.

The child died on the 25th, and on examination it was found that the pharynx terminated in a *cul de sac*; and that from the cardiac opening in the stomach, the œsophagus, after passing in the usual direction upwards for about 1½ inch, terminated in a cul de sac also. In the intervening space no thickened structure, resembling the remains of an œsophagus, could be traced. The muscles elevating the trachea and pharynx were complete, and the muscular structure of the pharynx and of a portion of the œsophagus was obvious.

The stomach and intestines were in a natural state, but extremely contracted, as might be supposed.—*London Lancet.*

---

*Serous Secretions.*—On the 13th of January of this year, M. Guerin presented a paper to the Royal Academy of Sciences of Paris, in which he proposed to establish as a fact, that atmospheric pressure performed an important part in the function of secretion from the serous membranes in the human body.—*Archiv. Gen. de Med.*

